# Hair cortisol as a marker of glucocorticoid replacement adequacy in adrenal insufficiency

**DOI:** 10.3389/fendo.2026.1765179

**Published:** 2026-04-01

**Authors:** Merve Korkmaz Yilmaz, Semih Tek, Gizem Karatas Kiliccioglu, Aysel Unver Ozkahraman, Mutlu Niyazoglu, Esra Hatipoglu

**Affiliations:** 1 Department of Endocrinology and Metabolism, University of Health Sciences, Basaksehir Cam and Sakura City Hospital, Istanbul, Türkiye; 2 Department of Medical Biochemistry, University of Health Sciences, Basaksehir Cam and Sakura City Hospital, Istanbul, Türkiye; 3 University of Health Sciences, Pituitary Disorders Research Center, Istanbul, Türkiye

**Keywords:** adrenal insufficiency, biomarker, hair cortisol, hydrocortisone replacement, metabolic outcomes, overreplacement, underreplacement

## Abstract

**Background:**

Achieving physiologic glucocorticoid replacement in adrenal insufficiency (AI) remains challenging, as both under- and over-replacement contribute to morbidity. Hair cortisol concentration (HCC) reflects cumulative cortisol exposure and may provide clinically relevant information beyond single-time-point assessments.

**Methods:**

In this cross-sectional study, 64 adults with hydrocortisone-treated AI and 64 matched healthy controls were evaluated. HCC was measured from the proximal 3-cm hair segment. Clinical, anthropometric, metabolic, and dosing parameters were analyzed. Patients were categorized as undertreated (VAS-fatigue or VAS-pain ≥7) or overtreated (hypertension, hyperglycemia, or ≥5% weight gain). Correlation, ROC, and multivariable regression analyses were performed.

**Results:**

HCC was higher in AI patients than controls (4.3 vs. 1.75 ng/g, p < 0.01). In AI, HCC was higher in primary than in secondary disease (6.5 vs. 3.8 ng/g; p = 0.037); this difference remained significant after excluding patients with congenital adrenal hyperplasia (13.8 vs. 3.8 ng/g; p < 0.01). HCC was positively correlated with BMI, waist circumference, blood pressure, and hydrocortisone dose, and inversely correlated with fatigue, pain, and therapy duration (all p < 0.05). In multivariable analysis, AI subtype remained independently associated with HCC. When the AI subtype was excluded from the model, hydrocortisone dose emerged as an independent predictor. HCC demonstrated excellent discrimination for severe fatigue (AUC 0.906) and pain (AUC 0.898), and good performance for systolic hypertension (AUC 0.837). Undertreated patients had markedly lower HCC than overtreated patients (2.1 vs. 14.1 ng/g, p < 0.001).

**Conclusions:**

HCC reflects long-term glucocorticoid exposure in AI and differentiates patterns consistent with both underreplacement and overtreatment. These findings support HCC as a potential adjunctive tool for evaluating replacement adequacy. Prospective studies are needed to determine its role in dose optimization.

## 1 Introduction

Adrenal insufficiency (AI) is characterized by deficient glucocorticoid production resulting from primary adrenal failure or disruption of the hypothalamic–pituitary–adrenal (HPA) axis. Lifelong glucocorticoid replacement is required to prevent adrenal crisis; however, achieving physiologic cortisol exposure with standard hydrocortisone regimens remains a major clinical challenge (1, 2). Both under- and over-replacement contribute to substantial morbidity, yet therapeutic decisions frequently rely on subjective symptoms and nonspecific metabolic markers (3). Conventional biochemical indices, such as serum cortisol, ACTH, or urinary free cortisol, provide only transient snapshots of glucocorticoid availability and fail to reflect cumulative cortisol exposure due to circadian variation, dosing peaks, and assay timing (4).

Hair cortisol concentration (HCC) has emerged as a promising biomarker that integrates systemic cortisol exposure over extended periods. Given that scalp hair grows approximately 1 cm per month, analysis of the proximal 3 cm segment reflects the preceding three months of cumulative cortisol secretion (5). HCC has been extensively validated in chronic stress research, with meta-analyses demonstrating robust associations between psychological stressors and elevated hair cortisol (6–8). In endogenous hypercortisolism, multiple studies have reported markedly elevated HCC levels in patients with Cushing’s syndrome, with strong correlations with disease activity and normalization upon remission (9–11). Beyond endogenous cortisol excess, HCC has also been used to assess exogenous glucocorticoid exposure. Systemic hydrocortisone replacement has been associated with increased HCC levels, reflecting cumulative cortisol exposure, whereas local corticosteroid exposure (e.g., topical formulations) may reduce HCC by suppressing hypothalamic–pituitary–adrenal axis activity. Currently available hair cortisol assays are analytically specific for cortisol (hydrocortisone) and cortisone and do not quantify other synthetic glucocorticoids administered orally or locally. Accordingly, interpretation of HCC must be considered in the context of the specific glucocorticoid administered and the analytical target of the assay (12–14).

Despite the rapidly expanding literature on HCC in both endogenous and exogenous hypercortisolism, evidence regarding its utility in AI remains scarce. The few adult studies available suggest that HCC reflects not only the prescribed hydrocortisone dose but also individual metabolic and anthropometric factors—such as BMI and age—that influence glucocorticoid metabolism and incorporation into hair (15, 16). Pediatric studies, particularly in congenital adrenal hyperplasia, further demonstrate that HCC increases in parallel with supraphysiologic glucocorticoid exposure, indicating that HCC can capture cumulative exogenous glucocorticoid burden even when dosing is intended to be physiologic (17). However, no prior research has examined whether long-term cortisol exposure, as quantified by HCC, corresponds to the metabolic and symptomatic indicators that clinicians routinely rely upon when assessing under- or over-replacement—such as weight changes, fatigue, pain, blood pressure abnormalities, or glycemic dysregulation. This represents a critical gap in the literature, especially given that treatment titration in AI continues to depend largely on subjective symptoms and short-term biochemical markers that do not accurately reflect cumulative glucocorticoid exposure. Whether HCC can discriminate clinical phenotypes of glucocorticoid insufficiency or excess - mirroring the real-world categories of undertreatment and overtreatment used in clinical practice - also remains unknown.

Given these gaps, the present study aimed to (i) compare HCC in adults with hydrocortisone-treated AI versus healthy controls; (ii) assess associations between HCC and clinical and metabolic variables within the AI group; and (iii) examine differences in HCC between primary and secondary AI. Additionally, by integrating a long-term cortisol biomarker with detailed clinical phenotyping, this study evaluates whether HCC may serve as an objective indicator of cumulative glucocorticoid exposure and symptom burden in adults with adrenal insufficiency.

## 2 Methods

### 2.1 Study design and participants

This observational, cross-sectional study included 64 adults (≥18 years) with AI receiving hydrocortisone replacement therapy and 64 age- and sex-matched healthy controls, recruited consecutively from the Endocrinology Outpatient Clinic of Basaksehir Cam and Sakura City Hospital between November 2024 and November 2025. Written informed consent was obtained from all participants, and the study protocol was approved by the institutional ethics committee (Approval No. 2024-246; November 27, 2024).

The diagnosis of AI was based on the clinical presentation, supported by biochemical testing, including morning serum cortisol and ACTH measurements, and by dynamic adrenal function assessment when indicated. Primary adrenal insufficiency was defined by a morning serum cortisol level <3 µg/dL in the presence of elevated ACTH concentrations. Secondary adrenal insufficiency was defined by a morning serum cortisol level <3 µg/dL with inappropriately low or normal ACTH levels. In equivocal cases, a standard 1-µg or 250-µg ACTH (Synacthen) stimulation test was performed; a peak serum cortisol <18 µg/dL was considered diagnostic. Congenital adrenal hyperplasia (CAH) cases were classified within the primary adrenal insufficiency group. All patients with primary adrenal insufficiency were receiving stable fludrocortisone replacement therapy, with no dose adjustments during the three months preceding hair sampling.

Healthy controls had no known endocrine disease and underwent standardized evaluation of hypothalamic–pituitary–adrenal (HPA) axis function. Individuals with a morning (08:00) serum cortisol >18 µg/dL were considered to have normal adrenal reserve. Participants with cortisol <3 µg/dL were excluded, whereas those with intermediate values underwent a 1 µg ACTH stimulation test; a peak cortisol ≥18 µg/dL at 30 or 60 minutes was required for inclusion.

Exclusion criteria for both groups included pregnancy, active infection, chronic inflammatory disease, major psychiatric disorders known to influence HCC (e.g., major depressive disorder, bipolar disorder, PTSD), use of any exogenous glucocorticoids other than prescribed hydrocortisone replacement (including oral, inhaled, topical, or injectable formulations), hair length <3 cm, scalp disorders, recent chemotherapy, or incomplete clinical data. Because hair cortisol reflects approximately three months of cumulative exposure, individuals who had experienced an adrenal crisis or received stress-dose or parenteral glucocorticoids within this period were also excluded. Patients with documented non-adherence to hydrocortisone therapy were excluded to ensure accurate assessment of replacement adequacy.

### 2.2 Clinical and laboratory assessment

For each participant, demographic characteristics (age, sex), anthropometric measurements (weight, height, BMI, and waist circumference), and disease-related variables (etiology of AI, disease duration, and daily hydrocortisone dose) were recorded. The mean daily hydrocortisone dose over the preceding three months was determined from medical records and verified through patient-reported treatment stability during that period. Blood pressure, including systolic blood pressure (SBP) and diastolic blood pressure (DBP), was measured in the seated position after at least five minutes of rest using an automated sphygmomanometer. Fasting plasma glucose (FPG) and glycated hemoglobin (HbA1c) were obtained following an overnight fast. Symptom burden was assessed using 10-cm Visual Analog Scales (VAS) for fatigue and pain, which have established validity in international populations, and recent weight gain (≥5% increase in body weight over the preceding 3 months) was documented (18, 19). Hair-care practices with the potential to influence cortisol deposition—including washing frequency, dyeing, and chemical treatments such as perms, keratin treatments, chemical straightening/relaxers, and bleaching—were recorded using a standardized checklist within the preceding three months.

### 2.3 Hair sample collection and cortisol measurement

Hair samples were collected from the posterior vertex region of the scalp, a site known to exhibit minimal intra-individual variability. A pencil-width section of hair (approximately 100 strands) was isolated and cut as close to the scalp as possible using clean surgical scissors, taking care to avoid skin irritation or blood contamination. The scalp-proximal end was immediately identified and labeled on the aluminum foil at the time of collection to preserve the segment’s orientation during storage. Each sample was wrapped in aluminum foil, placed in a sealed envelope, and stored at room temperature protected from sunlight until analysis. For cortisol assessment, only the proximal 3-cm segment—corresponding to approximately three months of cumulative cortisol exposure assuming a growth rate of 1 cm per month—was isolated and weighed (20).

Sample preparation followed a modified protocol based on the method described by Sauvé et al. A minimum of 40 mg of hair was cut into approximately 1-cm pieces and transferred into glass vials (21). Samples were washed with isopropanol and incubated in methanol at 52 °C for 16 hours to extract cortisol. The methanol extract was transferred to fresh tubes and evaporated to dryness using vacuum centrifugation over approximately four hours. The dried residue was reconstituted in phosphate-buffered saline (PBS) to a predetermined volume for immunoassay analysis.

Hair cortisol concentration was quantified using an electrochemiluminescence immunoassay (ECLIA) adapted from a validated method developed initially for serum and salivary cortisol. Measurements were performed using the Elecsys Cortisol II kit (Roche Diagnostics, Mannheim, Germany) on the Cobas e801 analytical platform. According to the manufacturer, the assay has a limit of quantification of 0.109 µg/dL and a measuring range of 0.054–63.4 µg/dL. Cortisol mass (ng) was calculated by multiplying the measured concentration by the reconstitution volume, and results were expressed as nanograms of cortisol per gram of hair.

### 2.4 Diagnostic criteria for undertreatment and overtreatment

Patients were categorized as undertreated if they reported severe symptoms, defined as VAS-fatigue or VAS-pain scores ≥7. Overtreatment was defined by the presence of at least one cardiometabolic criterion: systolic blood pressure ≥140 mmHg, diastolic blood pressure ≥90 mmHg, HbA1c ≥6.5%, fasting plasma glucose ≥126 mg/dL, or ≥5% recent weight gain. These definitions were based on established clinical thresholds to ensure independent assessment of glucocorticoid replacement status.

### 2.5 Statistical analysis

Statistical analyses were performed using IBM SPSS Statistics version 25.0. Normality was assessed using the Shapiro–Wilk test. Continuous variables are presented as mean ± SD or median (IQR), as appropriate, and categorical variables as number (percentage).

Univariate analyses were first conducted to examine associations between HCC and clinical variables. Between-group comparisons were performed using the independent t-test or Mann–Whitney U test for continuous variables and χ² or Fisher’s exact tests for categorical variables. Correlations between HCC and continuous clinical parameters were assessed using Spearman’s correlation analysis.

Variables showing clinical relevance and/or significant associations in univariate analyses were subsequently entered into a multivariable linear regression model to identify independent predictors of HCC. These included age, sex, BMI, waist circumference, AI subtype (primary vs. secondary), hydrocortisone dose parameters, total duration of therapy, and hair-related variables. Cardiometabolic parameters (e.g., systolic and diastolic blood pressure) and symptom scores (VAS-fatigue and VAS-pain) were excluded from the regression model, as they were considered downstream clinical manifestations of glucocorticoid exposure rather than independent determinants of HCC. Model assumptions were verified prior to analysis. To address potential collinearity among hydrocortisone dosing variables, additional regression models were constructed excluding either the 3-month mean dose or AI subtype. These sensitivity analyses were performed to evaluate the independent contribution of dosing parameters to HCC.

To provide a biologically anchored reference framework, an additional exploratory analysis was performed using the 2.5th and 97.5th percentiles of hair cortisol concentrations (HCC) derived from the healthy control group. Based on these thresholds, AI patients were reclassified as undertreated (HCC < 2.5th percentile), adequately treated (HCC between the 2.5th and 97.5th percentiles), or overtreated (HCC > 97.5th percentile). Comparisons of hydrocortisone dose parameters across percentile-defined categories were performed using the Kruskal–Wallis test. Logistic regression analysis was additionally performed to assess the association between hydrocortisone dose and percentile-defined overtreatment.

Receiver operating characteristic (ROC) analyses were performed separately to evaluate the discriminative ability of HCC in distinguishing clinically defined undertreatment from overtreatment. Optimal cutoffs were determined using the Youden index.

## 3 Results

### 3.1 Participant characteristics

The study included 128 participants: 64 patients with AI and 64 age- and sex-matched healthy controls. Demographic and anthropometric characteristics were comparable between groups (**Table 1**). Patients with AI exhibited significantly higher fatigue and pain scores and a greater prevalence of recent weight gain and diabetes mellitus compared with controls (**Table 1**). HbA1c levels were higher in the AI group, whereas fasting glucose levels were similar between groups. Systolic blood pressure did not differ significantly; however, diastolic blood pressure was modestly higher in patients with AI (**Table 1**).

**Table 1:** Comparison of sociodemographic and clinical variables.

Variables	Adrenal insufficiency (n = 64)	Healthy controls (n = 64)	p-value
Age (years), mean ± SD	39.7 ± 15	39.6 ± 12	0.99^a^
Gender, n(%)			
Female	48 (75)	50 (78)	0.67^b^
Male	16 (25)	14 (22)	
BMI (kg/m²), mean ± SD	27.3 ± 5.7	27 ± 6.9	0.82^a^
Waist circumference (cm)	93.8 ± 18.6	93.9 ± 20.4	0.97^a^
Recent weight gain (past 3 months), n(%)			
No	56 (88)	64 (100)	0.007^c^
Yes	8 (12)	0 (0)	
VAS-Fatigue (0–10), median (IQR)	6 (4–7)	2 (1.5–3)	<0.01^d^
VAS- Pain (0-10), median (IQR)	3 (2–5)	1 (1–2)	<0.01^d^
Diabetes mellitus, n(%)			
No	57 (89)	64 (100)	0.003^c^
Yes	7 (11)	0 (0)	
Current Hba1c (%)	5.7 ± 0.9	5 ± 0.7	<0.01^a^
Current fasting blood glucose (mg/dL)	94 ± 26.6	93.5 ± 10.7	0.89^a^
Systolic blood pressure (mmHg), mean ± SD	116 ± 15.1	112 ± 9.6	0.07^a^
Diastolic blood pressure (mmHg), mean ± SD	80 ± 8.2	77.2 ± 8.3	0.039^a^
Hair dye use, n(%)			
No	59 (92)	56 (88)	0.56^b^
Yes	5 (8)	8 (12)	
Hair chemical treatment, n(%)			
No	64 (100)	64 (100)	NA
Yes	0 (0)	0 (0)	
Hair washing frequency (per week)	3.3 ± 1.7	3.3 ± 1.5	0.97^a^
Hair cortisol (ng/g), median (IQR)	4.3 (1.8 – 13.6)	1.75 (0.98 – 4.1)	<0.01^d^

HC, Hydrocortisone, ^a^Independent t-test, ^b^Pearson chi-square test, ^c^Fisher’s exact test; ^d^Mann–Whitney U Test, p < 0.05 was considered statistically significant, NA: Not applicable.

### 3.2 Hair cortisol concentrations across adrenal insufficiency subtypes and healthy controls

Among the 64 patients with AI, 31 had primary and 33 had secondary adrenal insufficiency. Of the 31 patients with primary AI, 24 (77%) had Addison’s disease, and 7 (23%) had congenital adrenal hyperplasia, whereas all 33 patients with secondary AI had central adrenal insufficiency. Hair cortisol concentrations (HCC) were significantly higher in AI patients than in healthy controls (median [IQR]: 4.3 [1.8–13.6] vs. 1.75 [0.98–4.1] ng/g; p < 0.01). The distribution of HCC in patients showed greater variability than in controls. Within the AI group, patients with primary adrenal insufficiency had higher median HCC values compared with secondary adrenal insufficiency (6.5 [3.3–21.6] vs. 3.8 [1.3–9.3] ng/g; p = 0.037) (**Table 2**). In a sensitivity analysis excluding patients with congenital adrenal hyperplasia, hair cortisol concentrations remained significantly higher in patients with Addison’s disease compared with secondary adrenal insufficiency (median 13.8 vs. 3.8 ng/g; p < 0.01).

**Table 2:** Comparison of hair cortisol levels by primary/secondary adrenal insufficiency and underlying etiologies.

Variables	Primary AI (n=31)	Secondary AI (n=33)	p-value
Total duration of HC therapy (years)	3 (0.7–10)	1 (0.4–6)	0.29
Daily HC dose of past 3 months (mg/day)	20 (15–27.5)	15 (10–20)	0.003
Current daily HC dose (mg/day)	20 (15–27.5)	15 (10–20)	0.002
Hair cortisol (ng/g)	6.5 (3.3–21.6)	3.8 (1.3–9.3)	0.037

Values are presented as median (interquartile range). Group differences were assessed using the Mann–Whitney U test; p < 0.05 was considered statistically significant.

### 3.3 Correlations between hair cortisol and clinical variables

**Figure 1:**
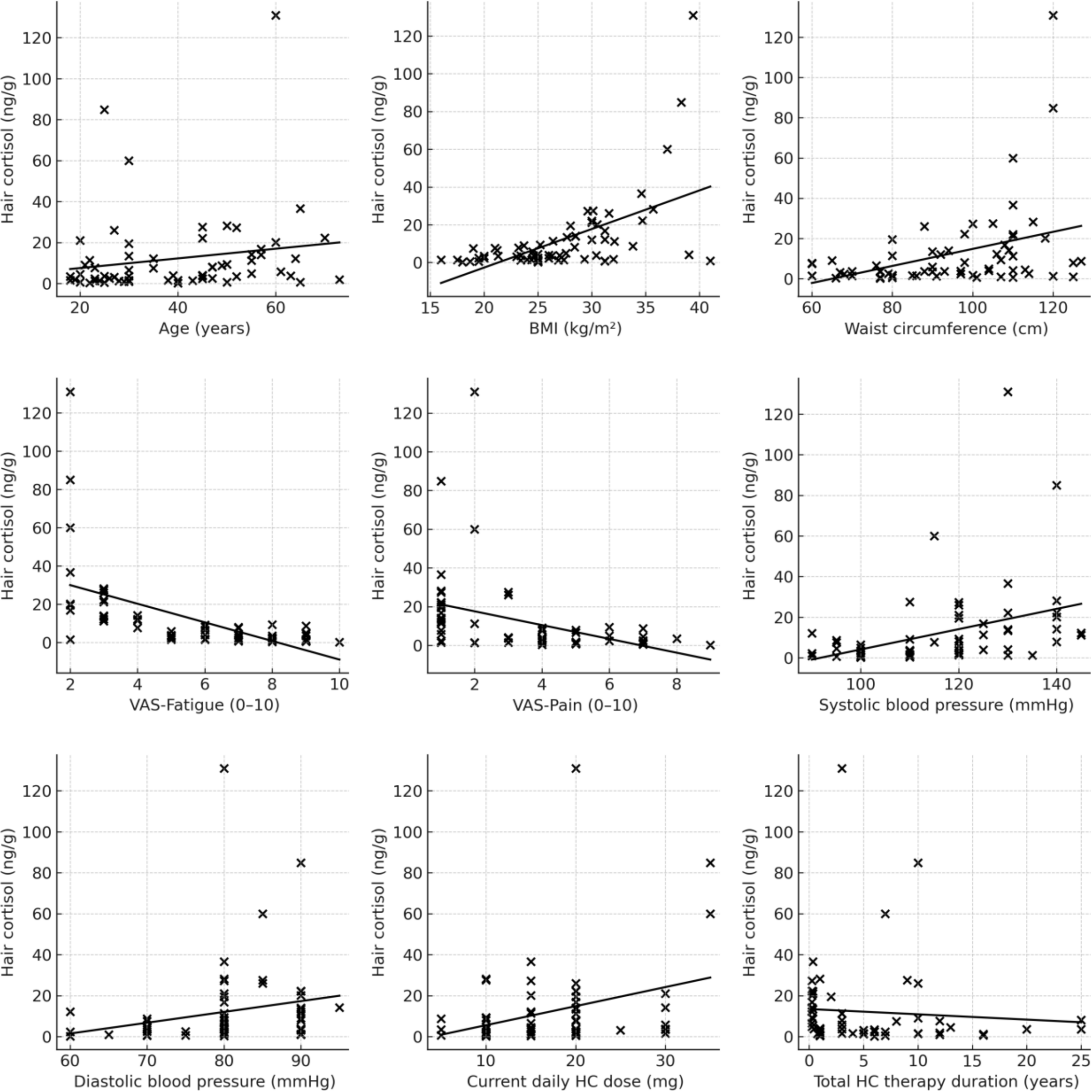
Correlation analyses between hair cortisol levels and clinical variables in patients with adrenal insufficiency. Spearman correlation coefficients were as follows: age (ρ = 0.323, p = 0.009), body mass index (ρ = 0.546, p < 0.001), waist circumference (ρ = 0.394, p = 0.001), fatigue score (ρ = –0.744, p < 0.001), pain score (ρ = –0.639, p < 0.001), systolic blood pressure (ρ = 0.502, p < 0.001), diastolic blood pressure (ρ = 0.387, p = 0.002), current daily hydrocortisone dose (ρ = 0.257, p = 0.041), and total duration of hydrocortisone therapy (ρ = –0.343, p = 0.006).

Correlation analyses were performed within the AI group. Statistically significant negative correlations between HCC and fatigue (ρ = –0.744; p < 0.001) and pain scores (ρ = –0.639; p < 0.001) were observed. Statistically significant positive correlations of HCC were found with age (ρ = 0.323; p = 0.009), BMI (ρ = 0.546; p < 0.001), waist circumference (ρ = 0.394; p = 0.001), systolic blood pressure (ρ = 0.502; p < 0.001), diastolic blood pressure (ρ = 0.387; p = 0.002), current daily hydrocortisone dose (ρ = 0.257; p = 0.041), and mean daily dose over the past 3 months (ρ = 0.284; p = 0.023). A statistically significant negative correlation was observed between HCC and total duration of hydrocortisone therapy (ρ = –0.343; p = 0.006) (**Figure 1**). No significant differences in HCC were observed according to sex, hair dye use, chemical treatment, washing frequency, glycemic markers, or diabetes status (all p > 0.05).

HCC values were comparable between participants with and without recent weight gain (8.3 vs. 4.0 ng/g; p = 0.48). Similarly, the current mean daily hydrocortisone dose did not differ between individuals reporting recent weight gain and those without (15.0 vs. 17.5 mg; p = 0.19).

### 3.4 Multivariable linear regression

**Figure 2:**
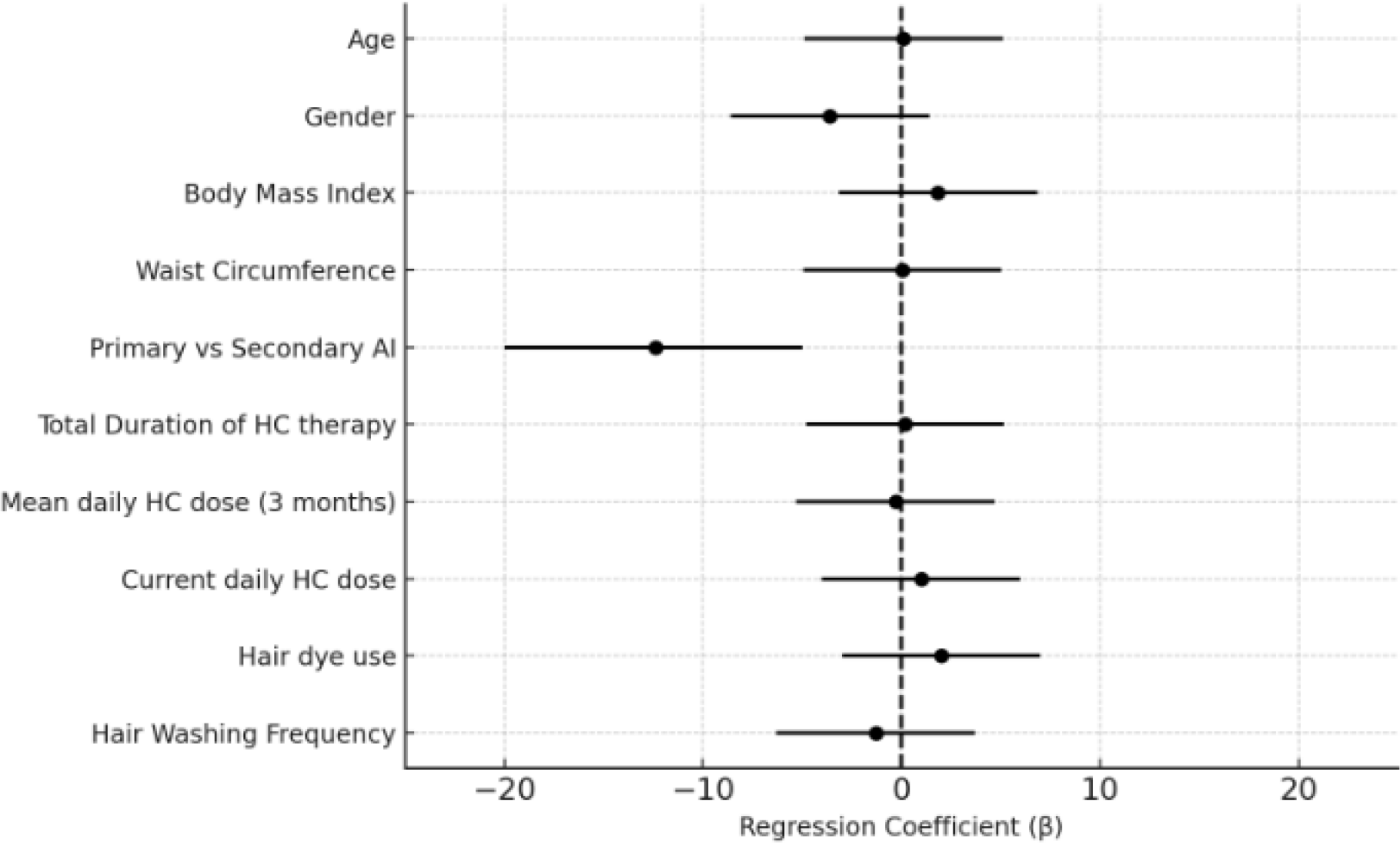
Multivariable linear regression analysis of factors associated with hair cortisol concentration. Multivariable regression analysis showed that only the distinction between primary and secondary AI (β = –12.4, p = 0.01) was independently associated with hair cortisol concentration.

In the primary multivariable linear regression model, AI subtype was the only independent predictor of hair cortisol concentration. HCC was significantly higher in patients with primary adrenal insufficiency compared with those with secondary AI (β = –12.4, p = 0.01) (**Figure 2**). Age, sex, BMI, waist circumference, hydrocortisone dose parameters, total duration of therapy, and hair-care practices were not significant predictors (all p > 0.05). The final model explained 51% of the variance in HCC (R² = 0.514, p < 0.001).

Given the potential interrelationship among hydrocortisone dosing variables and AI subtype, additional analyses were performed to assess model robustness. In a revised model excluding the 3-month mean hydrocortisone dose to reduce collinearity, the current daily hydrocortisone dose emerged as an independent predictor of HCC (β = 0.70, p = 0.039). Furthermore, in a sensitivity analysis excluding the AI subtype from the model, hydrocortisone dose parameters showed significant independent associations with HCC (current daily dose: β = 0.97, p = 0.003; 3-month mean dose: β = 0.97, p = 0.004, when modeled separately).

### 3.5 ROC analyses for identifying glucocorticoid undertreatment

ROC analyses were performed to evaluate whether HCC could identify severe symptom levels. For severe fatigue, the model yielded an AUC of 0.906 (95% CI: 0.84–0.96) with an optimal HCC cutoff of 4.9 ng/g, corresponding to a sensitivity of 93.1%, specificity of 80.0%, a positive predictive value of 79.4%, and a negative predictive value of 93.3%. For severe pain, the AUC was 0.898 (95% CI: 0.80–0.97), and the optimal HCC cutoff was 3.5 ng/g, achieving a sensitivity of 100%, specificity of 72.2%, a positive predictive value of 40.0%, and a negative predictive value of 100%.

### 3.6 ROC analyses for identifying glucocorticoid excess

ROC analyses were performed to evaluate the ability of HCC to discriminate clinical features associated with glucocorticoid excess. Using predefined cardiometabolic thresholds, HCC demonstrated its strongest discriminatory performance for elevated systolic blood pressure (AUC 0.837, 95% CI 0.74–0.93). An optimal cutoff of 7.9 ng/g yielded high sensitivity (100%) with moderate specificity (67.9%). Discriminatory capacity was lower for diastolic hypertension (AUC 0.626, 95% CI 0.51–0.74) and for hyperglycemic markers (HbA1c ≥6.5% or FPG ≥126 mg/dL; AUC 0.608, 95% CI 0.49–0.72). Across outcomes, a similar HCC threshold of approximately 8 ng/g was observed.

### 3.7 Exploratory reclassification of AI patients using control-derived HCC percentiles

Using control-derived HCC percentiles (2.5th: 0.56 ng/g; 97.5th: 15.08 ng/g), AI patients were reclassified as undertreated (n=3, 5%), adequately treated (n=47, 73%), or overtreated (n=14, 22%). The hydrocortisone dose tended to be higher in the overtreatment group than in the adequate group; however, the difference did not reach statistical significance (**Table 3**). Logistic regression analysis showed a positive but non-significant association between hydrocortisone dose and percentile-defined overtreatment (OR 1.08 per 1 mg increase; 95% CI 0.99–1.18; p=0.06). These percentile-based classifications were considered exploratory and are distinct from the clinically defined treatment categories presented above.

**Table 3:** Percentile-based reclassification of AI patients.

Variables	Undertreated (n=3)	Adequate (n=47)	Overtreated (n=14)	p-value
Hair cortisol (ng/g)	<0.56	0.56–15.08	>15.08	–
Current daily HC dose (mg)	15 (12.5–17.5)	15 (10–20)	20 (15–20)	0.20
Mean daily HC dose (mg) (last 3 months)	13.3 (11.7–14.2)	15 (10–20)	20 (15–20)	0.11
Addison, n	1	18	5	–
CAH, n	0	6	1	–
Secondary AI, n	2	23	8	–

Values are presented as median (IQR). Percentile thresholds were derived from controls. Kruskal–Wallis was used for dose comparisons. Hair cortisol was the defining variable and was not statistically compared.

### 3.8 Comparison of clinically defined under- and over-treatment groups

In analyses based on clinically defined treatment categories, glucocorticoid dosing parameters were higher in the overtreatment group (**Table 4**). Hair cortisol concentrations were significantly higher in overtreated patients than in undertreated individuals (median 14.1 vs. 2.1 ng/g; p < 0.001).

**Table 4:** Comparison of glucocorticoid dosing parameters and hair cortisol concentrations between under- and overtreatment groups.

Variables	Under-treatment (n = 22)	Over-treatment (n = 14)	p-value
Current daily HC dose (mg)	15.0 (10 – 18.8)	20 (15 – 20)	0.05
Mean daily HC dose (mg) (last 3 months)	12.45 (10 – 16.9)	20 (15 – 20)	0.02
Total duration of HC therapy (years)	1 (1 – 6.8)	0.33 (0.25 – 2.5)	0.03
Hair cortisol concentration (ng/g)	2.1 (0.8 – 3.8)	14.1 (11.5 – 22.2)	<0.001

Values are presented as median (interquartile range). Group differences were assessed using the Mann–Whitney U test. The undertreatment group is defined by VAS-fatigue or VAS-pain ≥7, and the overtreatment group by SBP ≥140 mmHg, DBP ≥90 mmHg, HbA1c ≥6.5%, FPG ≥126 mg/dL, or ≥5% weight gain.

## 4 Discussion

In this cross-sectional study, we demonstrate that adults with adrenal insufficiency receiving hydrocortisone replacement exhibit substantially higher long-term cortisol exposure, as reflected by elevated hair cortisol concentrations (HCC). Importantly, these findings apply specifically to hydrocortisone replacement, as hair cortisol assays quantify incorporated cortisol (hydrocortisone) and not other synthetic glucocorticoids. Within the patient group, higher HCC values were consistently associated with metabolic and cardiovascular strain, including increased BMI, waist circumference, and systolic blood pressure, whereas lower HCC values were associated with more severe fatigue and pain. These findings support the role of HCC as a marker of glucocorticoid exposure.

Patients with adrenal insufficiency demonstrated substantially higher HCC than healthy controls (4.3 vs. 1.75 ng/g, p < 0.01), and individuals with primary AI showed greater cumulative exposure than those with secondary AI (6.5 vs. 3.8 ng/g, p = 0.037), consistent with previous observations (15–17). Higher HCC was associated with increased BMI, waist circumference, and systolic blood pressure, patterns consistent with known metabolic and vascular effects of glucocorticoid excess (22–27). Conversely, lower HCC correlated with fatigue and pain, in line with evidence linking reduced glucocorticoid signaling to symptom burden (25, 28, 29).

In addition to associations with symptoms and metabolic parameters, the relationship between HCC and hydrocortisone dosing further supports its role as a marker of glucocorticoid exposure. Hydrocortisone dose was positively associated with HCC in univariable analyses, consistent with prior evidence that hair glucocorticoids integrate systemic exposure over time (5–8). In multivariable models including AI subtype, dose effects were attenuated, likely due to collinearity with disease etiology; however, dose became significant when subtype was excluded, indicating that treatment intensity contributes meaningfully to cumulative exposure. Patients with primary AI exhibited higher HCC levels than those with secondary AI, a difference that may partly reflect higher replacement doses in this group. Given the cross-sectional design, the independent contributions of etiology and dosing strategies cannot be fully disentangled; however, similar findings after exclusion of congenital adrenal hyperplasia support the robustness of this association. Associations with treatment duration and age should also be interpreted cautiously. Overall, these findings reinforce HCC as an integrative marker of long-term glucocorticoid exposure in patients receiving hydrocortisone replacement.

Beyond metabolic and hemodynamic associations, HCC demonstrated strong discriminative performance for symptom severity, particularly fatigue and pain. ROC analyses yielded AUC values of 0.906 for severe fatigue and 0.898 for severe pain, indicating excellent discrimination. Optimal thresholds for identifying undertreatment were lower than those observed for cardiometabolic indicators (3.5–4.9 ng/g vs. approximately 8 ng/g) and showed high sensitivity and negative predictive value. These findings suggest that reduced long-term cortisol exposure is closely associated with greater symptom burden. HCC may therefore serve as a marker of glucocorticoid underreplacement.

Among indicators of glucocorticoid excess, HCC showed its strongest discriminative performance for systolic hypertension (AUC 0.837), whereas discrimination was weaker for diastolic hypertension and glycemic parameters (AUC ~0.60). The optimal HCC cutoff was similar across outcomes (approximately 8 ng/g), suggesting a threshold beyond which cardiometabolic effects of glucocorticoid exposure become more evident. These findings support the potential utility of HCC in identifying glucocorticoid-related increases in systolic blood pressure, whereas metabolic markers show more limited performance.

Although ROC analyses were conducted separately for under- and overtreatment outcomes, optimal HCC thresholds clustered within a limited range (3.5–4.9 ng/g for symptoms and approximately 8 ng/g for cardiometabolic indicators). HCC values between approximately 5 and 8 ng/g may represent a potentially optimal exposure range in hydrocortisone-replaced adrenal insufficiency; however, this observation is exploratory and requires validation in larger cohorts before clinical application.

A significant novel aspect of this study is the demonstration that HCC not only correlates with continuous clinical variables but also categorically distinguishes undertreatment and overtreatment phenotypes—two constructs central to real-world glucocorticoid management. Patients classified as undertreated (VAS ≥7) exhibited markedly lower HCC values, while those classified as overtreated (hypertension, hyperglycemia, or ≥5% weight gain) showed substantially higher concentrations. The magnitude of the difference between groups (median 2.1 ng/g vs 14.1 ng/g; p < 0.001) and the minimal overlap in distribution underscore the discriminatory strength of HCC as a biomarker of glucocorticoid adequacy.

The exploratory percentile-based reclassification using control-derived HCC thresholds yielded a broadly consistent distribution, with approximately one-fifth of patients exceeding the upper biological reference limit. Although the hydrocortisone dose showed a dose-dependent trend toward higher values in the overtreatment group, the difference did not reach statistical significance. These findings suggest that biologically anchored classification does not materially alter the overall interpretation derived from clinically defined treatment groups. When percentile-based categories were stratified by etiology, no striking imbalance suggestive of systematic undertreatment or overtreatment within a specific subtype was observed. These findings should be interpreted cautiously, given the subgroup sizes.

The current study was not without certain limitations. The sample size was modest, and the cross-sectional design precludes causal inference. Although BMI was not used as a matching variable, it was included in multivariable analyses and did not materially alter the findings. Hydrocortisone dosing schedules were heterogeneous, and pharmacokinetic factors such as dose timing were not quantified. In addition, ROC-derived thresholds were derived from a relatively small cohort and require validation in larger longitudinal studies. Despite these limitations, the consistency of associations across metabolic, symptomatic, and exposure-related analyses supports HCC as a potential integrative biomarker of glucocorticoid replacement adequacy.

These findings suggest that hair cortisol concentration may provide additional insight into long-term cortisol exposure beyond single-time-point assessments and may help contextualize persistent symptom heterogeneity in adrenal insufficiency. By linking cumulative cortisol exposure to both metabolic strain and symptom burden—and demonstrating strong discrimination for severe fatigue and pain—this study highlights the potential value of incorporating long-term exposure measures alongside traditional clinical assessment. Given that routine biochemical monitoring is not standard practice in adrenal insufficiency, HCC may be most appropriately considered as an adjunctive tool in selected cases where treatment decisions remain uncertain. Future longitudinal studies in larger cohorts are required to validate these findings and define the role of extended cortisol exposure markers in clinical practice.

## 5 Conclusion

In conclusion, hair cortisol concentration provides insight into long-term glucocorticoid exposure in adrenal insufficiency and differentiates clinical patterns consistent with both insufficient and excessive replacement. Lower HCC values were associated with greater symptom burden, whereas higher values were linked to cardiometabolic strain. These findings indicate that HCC may complement clinical assessment in selected situations. Further longitudinal studies are needed to clarify its potential role in individualized glucocorticoid management.

## Data Availability

The original contributions presented in the study are included in the article/supplementary material. Further inquiries can be directed to the corresponding author.
